# Regional variation in obstetrical intervention for hospital birth in the Republic of Ireland, 2005–2009

**DOI:** 10.1186/1471-2393-12-123

**Published:** 2012-11-05

**Authors:** Jennifer E Lutomski, John J Morrison, Mona T Lydon-Rochelle

**Affiliations:** 1 National Perinatal Epidemiology Centre, Department of Obstetrics and Gynaecology, Cork University Maternity Hospital 5th floor, Wilton, Cork, Ireland; 2 Department of Obstetrics and Gynaecology, Clinical Science Institute, National University of Ireland, Galway, Ireland; 3 Department of Epidemiology and Public Health, University College Cork, Cork, Ireland

**Keywords:** Caesarean section, Clinical practice variations, Epidural anaesthesia, ICD-10, Hospital discharge data

## Abstract

**Background:**

Obstetrical interventions during childbirth vary widely across European and North American countries. Regional differences in intrapartum care may reflect an inpatient-based, clinician-oriented, interventional practice style.

**Methods:**

Using nationally representative hospital discharge data, a retrospective cohort study was conducted to explore regional variation in obstetric intervention across four major regions (Dublin Mid Leinster; Dublin Northeast; South; West) within the Republic of Ireland. Specific focus was given to rates of induction of labour, caesarean delivery, epidural anaesthesia, blood transfusion, hysterectomy and episiotomy. Logistic regression analyses were performed to assess the association between geographical region and interventions while adjusting for patient case-mix.

**Results:**

323,588 deliveries were examined. The incidence of interventions varied significantly across regions; the greatest disparities were observed for rates of induction of labour and caesarean delivery. Women in the South had nearly two-fold odds of having prostaglandins (adjusted OR: 1.75, 95% CI 1.68-1.82), whereas women in the West had 1.85 odds (95% CI 1.77-1.93) of artificial rupture of membrane. Women delivering in the Dublin Northeast, South and West regions had more than two-fold increased odds of elective caesarean delivery relative to women delivering in the Dublin Mid Leinster region. The Dublin Northeast region had the highest odds of emergency caesarean delivery (adjusted OR: 1.36; 95% CI: 1.31-1.40).

**Conclusions:**

Substantial regional variation in intrapartum care was observed within this small, relatively homogeneous population. The association of intervention use with region illustrates the need to encourage uptake of scientific based practice guidelines to better inform clinical judgment.

## Background

The adoption and adherence to strong evidence-based clinical guidelines have been shown to improve maternal and perinatal outcomes [1,2]. Yet, wide variation in obstetrical intervention practice during childbirth has persisted across European and North American countries [3-6]. Such regional differences in obstetric practice are not necessarily explained by differences in socioeconomic status, women’s preferences, or severity of co-morbidities. Rather, differences may reflect a more inpatient-based, clinician-oriented, interventional practice style.

The identification of regional differences in intrapartum intervention has obvious implications for policy implementation. However, to date, such research has not been undertaken in the Republic of Ireland, a country with a historical reputation for both good obstetric training and perinatal outcomes. For instance, despite its stringent abortion legislation, Republic of Ireland has relatively low rates of perinatal mortality [7]. Further, in 2006, Republic of Ireland reported the lowest preterm delivery rate in the European Union and Norway [8].

In this context, we used administrative hospital discharge data to explore rates of frequently performed intrapartum interventions across four major regions in Ireland over a five-year period. We hypothesised there would be minimal variation between regions given the country’s small jurisdiction (approximately four million residents), its limited number of maternity units (20 in total), and that fact that obstetric trainees typically rotate within this small network. We further examined temporal trends to assess if regional patterns in intrapartum intervention were persistent over time.

## Methods

### Data source and study cohort

Childbirth hospitalisation is the most frequently listed admission for any hospitalisation among all acute care public specialty hospitals in the Republic of Ireland [9]. Under the Health Service Executive (HSE) Maternal and Infant Scheme, all pregnant women ordinarily resident in the country receive universal coverage for pregnancy-related care and services from public Irish maternity hospitals [10].

Data for this retrospective population-based cohort study were obtained from the Hospital In-Patient Enquiry (HIPE) database. Maintained by the Economic and Research Institute, HIPE is the only national level source of acute public hospital discharge records and has been extensively used for analysis of non-obstetric health services trends [11-16]. Each year, HIPE collects data on approximately 73,000 childbirth discharges from 19 of the 20 Irish maternity units [9], and represents approximately 97% of all recorded births in the country [7]. Using the HIPE database, we identified all women who had a singleton live birth in hospital between January 1, 2005 and December 31, 2009 (N=323,588).

**Figure 1:**
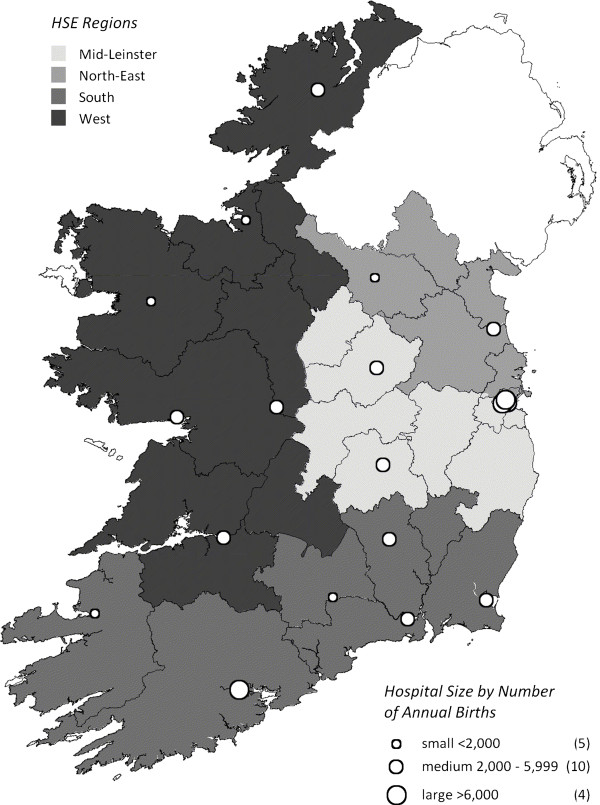
Map of 19 public maternity hospitals by Health Service Executive (HSE) region, Ireland.

Hospital-level data are not publically available in the HIPE dataset; the smallest geographical unit available for analysis is the HSE administrative hospital region. These regions, which acted as our primary predictor variable, are classified into four major areas (Dublin Mid Leinster; Dublin Northeast; South; West) and reflect both individual hospital and catchment areas. Based on government figures (data not published), the annual number of live births (rounded to the nearest 100) for each HSE region were approximately distributed as follows: (1) Dublin Mid Leinster has four hospitals with 22,000 live births (range 2,600-8,800); (2) Dublin Northeast has three hospitals with 14,000 live births (range 1,600-8,400); (3) the South has six hospitals with 17,700 live births (range 1,200-7,900); and (4) the West has six hospitals with 16,200 live births (range 1,300-5,200) (Figure 1). There are four large (≥8,000 deliveries per annum), tertiary referral hospitals in the Republic of Ireland, one is located in Dublin Northeast, two in Dublin Mid Leinster and one in the South.

The following measures for maternal characteristics and health service indicators were obtained from the HIPE database: maternal age; marital status; possession of a medical card (government assistance generally assigned to low-income individuals); length of stay; intensive care unit (ICU) admission; and type of discharge, which indicates if a patient was released to her home or other semi-permanent accommodation (i.e. convalescent home, foster care, or prison), absconded, or was transferred to an acute hospital, psychiatric ward, rehabilitation centre or hospice.

Incident cases of childbirth-related co-morbidities and interventions were identified using the HIPE dataset, which records up to 20 diagnoses and procedures coded according to the *International Statistical Classification of Diseases and Related Health Problems, Tenth Revision, Australian Modification* (ICD-10-AM). Maternal co-morbidities included only those reported during the hospitalisation for delivery. Co-morbidities were identified using a modified classification scheme as described by Lutomski *et al*[17]. The following ICD-10-AM codes were used for co-morbidity classifications: hypertensive disorders (I10-I12; I13.9; I15; O10; O11; O13-O16); gestational diabetes (O24.4); established diabetes (E10-14; O24.0,1,2,3); antepartum haemorrhage (O20.8,9; O45; O46); and placental disorders (O43.1,2,8,9; O44.1).

Since previous caesarean delivery may affect the decision for obstetrical intervention, we identified women with a uterine scar using the following two diagnostic codes: *Maternal care due to uterine scar from previous surgery (caesarean section)* (O34.2) and *Vaginal delivery following previous caesarean section* (O75.7). Parity was not available in the HIPE dataset.

Obstetrical procedures were identified by ICD-10-AM codes as well. In order to differentiate induction of labour from labour augmentation, we classified induction of labour using only induction-specific ICD-10-AM codes, including: *Medical induction of labour, oxytocin* (90465–00); *Medical induction of labour, prostaglandin* (90465–01); *Other medical induction of labour* (90465–02); *Surgical induction of labour by artificial rupture of membranes* (90465–03); *Other surgical induction of labour* (90465–04), or *Medical and surgical induction of labour* (90465–05). Mode of delivery was categorised as follows: elective caesarean (16520–00; 16520–02), emergency caesarean (16520–01; 16520–03), vacuum extraction (90469-00/01), or forceps extraction (e.g. breech, mid-cavity, high, low, rotation of fetal head and failed forceps) (90468-00/01/02/03/04/05; 90470-02/04). Measures of vacuum and forceps extraction were included whether these attempts had failed or succeeded.

Other interventions included epidural/spinal anaesthesia (92506-sub-divisions; 92507-sub-divisions; 92508-sub-divisions; 92516–00), episiotomy (90472–00), blood transfusion (13706-01/02/03; 92060–00; 92062–00) and hysterectomy (35653-00/01/02/03/04).

### Statistical analysis

Since certain interventions may eliminate the possibility of others during the delivery continuum (i.e. elective caesarean delivery precludes induction of labour), we identified three distinct groups to allow for the most robust comparisons of interventions between regions. Group 1 comprised of all women (n=323,588), Group 2 comprised of women with emergency caesarean or vaginal deliveries (operative and non-operative) (n=286,495) and Group 3 comprised of only women with vaginal deliveries (operative and non-operative) (n=243,291). Theoretically all women are at risk for elective caesarean delivery, blood transfusion or hysterectomy, and therefore subsequent analyses for these interventions were conducted within Group 1. Since emergency caesarean delivery, operative vaginal delivery, induction of labour and epidural anaesthesia are only relevant for women who undertook a trial of labour, analyses for these interventions were conducted within Group 2. Lastly, episiotomy is only relevant for women with vaginal deliveries, and thus this intervention was analysed within Group 3.

Characteristics of the study population were described across HSE administrative regions using means, medians or proportions. Using the appropriate base population as described above, five-year intervention rates were derived. We further calculated annual incidence rates between 2005 and 2009 and evaluated regional temporal trends using the Cochrane-Armitage test. Temporal analysis was limited to induction, mode of delivery, epidural anaesthesia and episiotomy as these rates were the most stable across years and regions. Logistic regression analyses were conducted to examine the association between HSE region and obstetric interventions with adjustment for relevant risk factors using the largest HSE region (Dublin Mid Leinster) as the referent region. Multivariate analysis was not performed for hysterectomy due to small numbers. All statistical analyses were conducted using SAS version 9.2 (SAS Institute, Inc., Carey, NC, USA).

### Details of ethical approval

This study was exempt from University College Cork Clinical Research Ethics Committee review as it used publicly available, anonymised data (Ref. No. ECM4(g)05/08/08).

## Results

Over the five-year study period, there were 323,588 deliveries; 102,509 occurred in Dublin Mid Leinster, 65,667 in Dublin Northeast, 79,789 in the South and 75,623 in the West. Differences in patient characteristics were observed across the four regions (Table 1). The percentages of married women and of women who received government medical assistance were highest in the West (67.3% and 34.9% respectively). Dublin Mid Leinster reported the lowest percentage of maternal ICU admissions (0.1%). Antepartum haemorrhage was relatively similar across regions, except for the West, which was modestly lower (0.8%). Placental disorders were highest in Dublin Northeast (1.1%) and lowest in Dublin Mid Leinster (0.4%).

**Table 1:** Patient characteristics by Health Service Executive hospital region for singleton, live birth delivery hospitalisations (N=323,588), Republic of Ireland, 2005-2009

**Characteristic**	**Dublin Mid Leinster (n=102,509)**	**Dublin Northeast (n=65,667)**	**South (n=79,789)**	**West (n=75,623)**
Age, years [mean (SD)]	30.6 (5.7)	30.0 (5.7)	30.4 (5.6)	30.7 (5.7)
Married	65,079 (63.5)	38,898 (59.2)	48,886 (61.3)	50,903 (67.3)
Medical card possession^a^	12,497 (12.2)	10,828 (16.5)	8,847 (11.1)	26,414 (34.9)
Length of stay, days [median (IQR)]	3 (2–4)	3 (2–4)	3 (2–5)	3 (2–5)
ICU admission	99 (0.1)	437 (0.7)	128 (0.2)	177 (0.2)
Hospital transfer^b^	75 (0.1)	66 (0.1)	144 (0.2)	144 (0.2)
Previous caesarean delivery	6,608 (10.1)	10,956 (10.7)	8,804 (11.0)	7,502 (9.9)
Morbidities				
Hypertensive disorders	5,228 (5.1)	3,677 (5.6)	3,957 (5.0)	4,271 (5.7)
Gestational diabetes	1,773 (1.7)	1,515 (2.3)	861 (1.1)	951 (1.3)
Established diabetes	374 (0.4)	178 (0.3)	279 (0.4)	211 (0.3)
Antepartum haemorrhage	1,234 (1.2)	870 (1.3)	1,092 (1.4)	606 (0.8)
Placental disorders	385 (0.4)	736 (1.1)	455 (0.6)	342 (0.5)

Note: Values are N (%) unless otherwise indicated.

Abbreviation: SD, standard deviation; IQR, inter-quartile range; ICU, intensive care unit.

^a^ Possession of a medical card provides low-income individuals with select free health care services.

^b^ Includes transfers to acute hospital, psychiatric ward, rehabilitation centre or hospice.

Use of prostaglandin for induction of labour was most common in the South (8.9%), with the other regions ranging from 4.7% to 5.9% (Table 2). The percentage of women receiving oxytocin was highest in Dublin Mid Leinster (6.8%), whereas the remaining regions used oxytocin at a rate of 1.8%. Artificial rupture of membranes (AROM) rates ranged from 3.6% to 8.9%.

**Table 2:** Incidence of interventions by Health Service Executive hospital region for singleton, live birth delivery hospitalisations, Republic of Ireland, 2005-2009

	**Dublin Mid Leinster**	**Dublin Northeast**	**South**	**West**
**Induction of labour**				
Prostaglandin	5.2	4.7	8.9	5.9
Oxytocin	6.8	1.8	1.8	1.8
Artificial rupture of membrane	5.1	3.6	4.4	8.9
Other^a^	12.2	13.5	12.3	15.4
Total induction	27.6	23.3	26.9	31.3
**Pain relief**				
Epidural anaesthesia	56.3	52.5	56.6	48.4
**Mode of delivery**				
Elective caesarean^b^	8.1	12.3	13.3	13.4
Emergency caesarean	14.7	16.4	15.7	13.7
Vacuum^c^	13.2	18.1	16.2	14.8
Forceps^c^	5.8	4.2	4.8	2.7
Non-operative vaginal	68.3	63.9	65.0	69.8
**Select procedures**				
Blood transfusion^b^	1.2	1.4	1.0	0.6
Hysterectomy^b^	0.02	0.03	0.01	0.02
Episiotomy^d^	27.4	18.5	22.4	21.8

Note: Values are rates per 100 deliveries. Rates are based on emergency and vaginal (operative and non-operative) deliveries only (n=286,495) unless otherwise specified.

^a^ Includes medical and surgical methods (i.e. Bougie, cervical dilation and Foley’s catheter) not elsewhere specified.

^b^ Rate is based on all deliveries (n=323,588).

^c^ Rate includes failed attempts.

^d^ Rate is based on vaginal (operative and non-operative) deliveries only (n=243,291).

Incidence rates of caesarean and operative vaginal deliveries varied across regions. While the West reported the highest elective caesarean delivery rate (13.4%), this region also reported the lowest emergency caesarean delivery rate (13.7%). Dublin Mid Leinster had the lowest vacuum-assisted delivery rate (13.2%) and the highest forceps-assisted delivery rate (5.8%). Exclusive of elective repeat caesarean deliveries, the rate of non-operative vaginal deliveries was higher in Dublin Mid Leinster and the West (68.3% and 69.8% respectively) and lower in Dublin Northeast and the South (63.9% and 65.0% respectively).

Blood transfusion was lowest in the South (0.6%) and highest in Dublin Northeast (1.4%). The rate of episiotomy among vaginal deliveries ranged from 18.5% in Dublin Northeast to 27.4% in Dublin Mid Leinster.

**Figure 2:**
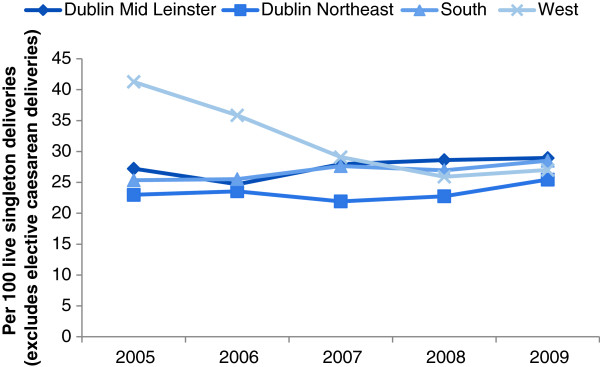
Temporal variation in induction (all forms) usage by Health Service Executive hospital region, Republic of Ireland.

**Figure 3:**
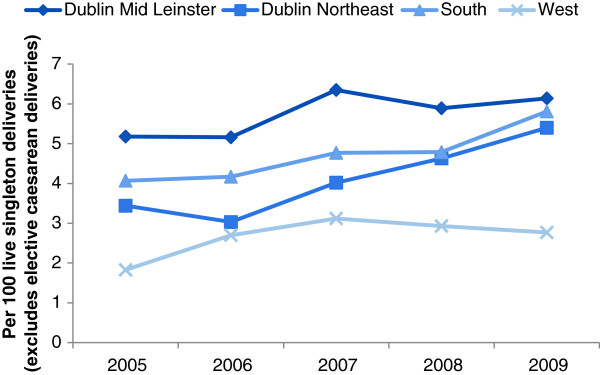
Temporal variation in forceps-assisted deliveries by Health Service Executive hospital region, Republic of Ireland.

**Figure 4:**
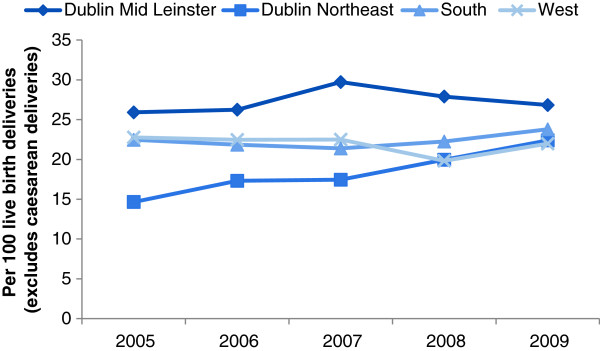
Temporal variation in episiotomy by Health Service Executive hospital region, Republic of Ireland.

Several temporal trends in obstetric intervention across HSE regions were observed between 2005 and 2009. While rates of induction of labour were fairly stable for Dublin Northeast, Dublin Mid Leinster and South regions, the West reported a significant decrease from 41.3% to 27.0% (test for trend, *p*-value<0.001) (Figure 2). Modest increases in forceps-assisted births were identified across Dublin Mid Leinster, Dublin Northeast and South regions (test for trend, *p*-value<0.001) (Figure 3). In contrast to the other regions, episiotomy rates in Dublin Northeast significantly increased from 14.7% in 2005 to 22.4% in 2009 (test for trend, *p*-value<0.001) (Figure 4); notably, there was no parallel increase in vaginal delivery rates over the time period. The incidence of caesarean delivery (both elective and emergency), vacuum-assisted delivery and epidural anaesthesia did not change substantially across any of the regions over the five-year period (data not shown).

Despite adjustment for risk factors, women in the South had nearly two-fold odds of having prostaglandins (adjusted OR: 1.75, 95% CI 1.68-1.82) (Table 3; Additional file 1: Table S1), which was significantly higher than the other regions. In comparison with the Dublin Mid Leinster region, the odds of receiving oxytocin were considerably lower in the three other regions. The odds of undergoing AROM for induction of labour in the West were 1.85 times greater (95% CI 1.77-1.93) than Dublin Mid Leinster.

**Table 3:** Adjusted odds ratio for select obstetric interventions by Health Service Executive hospital region, Republic of Ireland, 2005-2009

	**Dublin Mid Leinster**	**Dublin Northeast**	**South**	**West**
**Induction of labour**^a,b^				
Prostaglandin	1.00	0.86 (0.82-0.90)	1.75 (1.68-1.82)	1.12 (1.07-1.17)
Oxytocin	1.00	0.24 (0.22-0.25)	0.24 (0.23-0.26)	0.25 (0.23-0.26)
Artificial rupture of membrane	1.00	0.69 (0.65-0.72)	0.85 (0.81-0.89)	1.85 (1.77-1.93)
Other^c^	1.00	1.10 (1.06-1.13)	1.00 (0.97-1.03)	1.32 (1.28-1.36)
Total induction	1.00	0.77 (0.75-0.79)	0.95 (0.92-0.97)	1.20 (1.17-1.23)
**Pain relief**^a,d^				
Epidural anaesthesia	1.00	0.90 (0.88-0.92)	1.05 (1.03-1.07)	0.78 (0.77-0.80)
**Mode of delivery**				
Elective caesarean^b,e^	1.00	2.36 (2.26-2.46)	2.42 (2.33-2.52)	2.74 (2.63-2.86)
Emergency caesarean^a,f^	1.00	1.36 (1.31-1.40)	1.21 (1.18-1.25)	1.23 (1.19-1.27)
Vacuum^a,f^	1.00	1.54 (1.50-1.59)	1.26 (1.22-1.29)	1.36 (1.32-1.41)
Forceps^a,f^	1.00	0.76 (0.72-0.79)	0.81 (0.78-0.85)	0.54 (0.51-0.58)
Non-operative vaginal^a,f^	1.00	0.68 (0.67-0.70)	0.79 (0.77-0.81)	0.78 (0.76-0.80)
**Select procedures**				
Blood transfusion^b,e^	1.00	1.13 (1.03-1.23)	0.85 (0.78-0.93)	0.51 (0.46-0.57)
Episiotomy^g,h^	1.00	0.40 (0.39-0.42)	0.58 (0.57-0.60)	0.75 (0.72-0.77)

^a^ Based on emergency caesarean or vaginal (operative and non-operative) deliveries only (N=286,495).

^b^ Adjusted for age, marital status, medical card possession, previous caesarean delivery and co-morbidities.

^c^ Includes medical and surgical methods not elsewhere specified.

^d^ Adjusted for age, marital status, medical card possession, previous caesarean delivery, co-morbidities and induction of labour.

^e^ Based on all deliveries (N=323,588).

^f^ Adjusted for age, marital status, medical card possession, previous caesarean delivery, co-morbidities, induction of labour and epidural. The interaction term between induction of labour and epidural was also included in the model.

^g^ Based on vaginal deliveries only (N=243,291).

^h^ Adjusted for age, marital status, medical card possession, co-morbidities and operative vaginal delivery.

Women delivering in the Dublin Northeast, South and West regions had significantly increased odds of elective caesarean delivery relative to women delivering in the Dublin Mid Leinster region. Further, when consideration was given to the lower limits of the confidence intervals, the West clearly had a higher rate than all other regions (adjusted OR: 2.74; 95% CI: 2.63-2.86). The Dublin Northeast region had the highest odds of both emergency caesarean and vacuum-assisted delivery compared to all other regions. Relative to Dublin Mid Leinster, the odds of forceps delivery was lower for all other regions, particularly the West.

The odds of blood transfusion were highest in Dublin Northeast. Women delivering in Dublin Northeast had 0.40 (95% CI 0.39-0.42) the odds of having an episiotomy, which was the lowest among all four regions.

## Discussion

We found considerable variation in rates of obstetric intervention for induction of labour, operative vaginal delivery and epidural anaesthesia use across regions in the Republic of Ireland. Regional differences in obstetrical intervention have been reported within several large countries, including the United Kingdom [18], France [19], Canada [20] and the United States [21]. With approximately four million residents, the Republic of Ireland is considerably smaller than these countries; yet, clear patterns in regional variation persisted within this small, relatively homogenous population. Although the Republic of Ireland has a historically good reputation for obstetric practice, our findings support that individual clinician opinion and practice may exert a strong influence on obstetric intervention [21-23].

Recognising the need for consistent practice guidelines based on robust scientific evidence, obstetrical societies such as the Royal College of Obstetricians and Gynaecologists (RCOG) [24], the National Institute for Health and Clinical Excellence (NICE) [25,26], and the American College of Obstetricians (ACOG) [27-29], publish guidelines intended to offer guidance by providing information to help clinicians decide how best to care for women, allowing for clinical judgment and women’s preferences. Our findings highlight the need to improve the process of writing guidelines based on conclusive evidence for frequently practised obstetric interventions and to expand the uptake of guidelines to clinicians [30,31]. Importantly, intervention measures require more specification of why they were performed to avoid defining optimal rates based solely on threshold levels. These considerations are of particular importance for the Republic of Ireland, which is currently developing its own obstetric practice guidelines.

Despite increased awareness from the medical community and public that variations in the intrapartum intervention need to be addressed, interventions among this study cohort differed across region and over time. Several reasons may account for this observed variability, although our study cannot confirm which of these may be most important. Physicians and midwives practising in different regions might be more likely to favour discretionary interventions for which strong evidence is lacking [26,32]. Alternatively, women giving birth in different regions may be more likely to have underlying co-morbidities (i.e. hypertension) who are likely to benefit from interventions.

Nonetheless, our observations may have been a result of other baseline geographical differences that we were unable to explore in this analysis. For instance, parity, an important factor for obstetric intervention, was lacking in the dataset and could not be assessed across the regions. While seven of the 19 maternity units analysed in this study have published data on parity in their individual annual clinical reports, we did not have enough information to determine if parity significantly differed between regions. To minimise potential bias, we controlled for age, marital status, government medical assistance, maternal co-morbidities, previous caesarean delivery, induction of labour and epidural pain relief. Despite adjustment for these factors, observed differences in risk estimates between regions persisted.

To the knowledge of the authors, this study is the first to investigate regional variation in obstetrical intervention practices over a five-year period in the Republic of Ireland, and this work has two inherent strengths. Firstly, we reviewed hospital discharge records from 19 of the 20 Irish maternity hospitals, and therefore our analysis is nationally representative and captures nearly all births in the country (~97%). Secondly, unlike previous studies which have focused on a single intervention [18,19], we investigated multiple interventions by region. While our study refers to Irish practice, the level of detail included in our methodology coupled with the use of multivariate analysis to adjust for differences in patient case-mix ensure our findings are relevant to obstetric practice in other developed countries.

Our study has several important limitations. First, interventions and co-morbidities were derived from hospital discharge data, and thus observed regional variation may reflect differences in individual hospital reporting practice rather than true variations. Further, while reporting bias is an issue for registry datasets, diagnostic codes in particular may be more prone to underreporting [33]. For instance, our observed rates of gestational diabetes were notably lower than previous estimates from the UK (3.5%) [34] and Australia (4.6%) [35]. Still, examination of the quality of reporting of HIPE data indicates that the accuracy of reporting is very good [36-38]. Prior studies have reported that hospital discharge records are highly sensitive in detecting cases of caesarean section, hypertension and anaesthesia [39-41]. Reassuringly, our overall estimates of the incidence of several interventions are similar to those of previous studies [19,20], supporting the accuracy of the data.

A second limitation is the absence of some traditional predictors of obstetric interventions in the HIPE dataset, such as infant birth weight, estimated gestational age and obstetric history. While we were able identify women with a previous caesarean delivery, we could not discern the number or type (lower segment versus classical), both of which would impact on clinical decision-making.

Lastly, potential ecological fallacy is of note. Regional differences may in fact be distorting unit-specific differences. Due to data protection legislation, we were not permitted to extract unit-specific data. Therefore, we lacked information on location (i.e. metropolitan versus non-metropolitan), obstetric volume, organisational structure (i.e. availability of anaesthesia services) and treatment protocol, which inhibited a more thorough analysis of geographical differences.

Future studies addressing this question would be improved by developing maternally linked data sources in the Republic of Ireland, a common methodological approach employed in many countries (USA, Scotland, Norway, Australia) [42-45]. Linking hospital discharge records with birth notification forms would provide additional information on mothers (i.e. parity) and infants (i.e. gestational age). Such data would allow for better assessment of the effects of intrapartum intervention on perinatal outcomes. Due to the absence of such linked datasets, we can only refer to ecological evidence which, as aforementioned, has limitations in interpretability.

Notably, the national preterm birth rate is 6% [7] and national perinatal mortality rate is 6.9 per 1,000 live births and stillbirths [7], and these rates did not vary across HSE regions over the study period (data unpublished). While this observation may imply that differences in intrapartum interventions are unnecessary, the converse may in fact be true. Higher intervention rates in certain regions may be medically required to maintain good perinatal outcomes.

## Conclusion

In the absence of patient-related factors, hospital-level data and infant measures, regional differences in the incidence of intrapartum interventions herein are best viewed as suggestive rather than definitive evidence of differences in practice. Still, our investigation highlights substantial unexplained intrapartum regional variation in the care of women during hospital childbirth and suggests that initiatives could focus on specific processes of care to reduce such variation. The association of intervention use with region as well as temporal trends across regions illustrate the need to encourage uptake of scientific based practice guidelines for usage to better inform clinical judgment.

## Competing interests

All authors have nothing to declare.

## Authors' contribution

JEL contributed to the data analysis plan, undertook the data analysis and interpretation. JJM collaborated in formulating the study aims and interpretation of the data. MLR conceived and designed the study. All authors refined the ICD-10-AM coding schema, contributed to drafting the paper and approve its submission for publication. All authors read and approved the final manuscript.

## Pre-publication history

The pre-publication history for this paper can be accessed here:

http://www.biomedcentral.com/1471-2393/12/123/prepub

## Supplementary Material

Additional file 1 **Table S1.** Unadjusted odds ratios for select obstetric interventions by Health Service Executive hospital region, Republic of Ireland, 2005-2009. Click here for file
