# Supplementary material for: Regional variation in obstetrical intervention for hospital birth in the Republic of Ireland, 2005–2009
Source: BMC Pregnancy Childbirth. 2012 Nov 5;12:123. doi: 10.1186/1471-2393-12-123 (PMC3541199; doi:10.1186/1471-2393-12-123)
Supplement: Additional file 1 — Table S1. Unadjusted odds ratios for select obstetric interventions by Health Service Executive hospital region, Republic of Ireland, 2005-2009. [file 1471-2393-12-123-S1.doc]

Supplementary Table S1: Unadjusted odds ratios for select obstetric interventions by Health Service Executive hospital region, Republic of Ireland, 2005-2009

|  | Dublin Mid Leinster | Dublin Northeast | South | West |
| --- | --- | --- | --- | --- |
| **Induction of labour**^a^ |  |  |  |  |
| Prostaglandin | 1.00 | 0.90 (0.86-0.95) | 1.80 (1.73-1.87) | 1.14 (1.09-1.19) |
| Oxytocin | 1.00 | 0.25 (0.23-0.26) | 0.25 (0.24-0.27) | 0.26 (0.24-0.27) |
| Artificial rupture of membrane | 1.00 | 0.69 (0.65-0.72) | 0.85 (0.81-0.89) | 1.82 (1.75-1.89) |
| Other^b^ | 1.00 | 1.13 (1.09-1.16) | 1.02 (0.99-1.05) | 1.31 (1.28-1.35) |
| Total induction | 1.00 | 0.80 (0.78-0.82) | 0.97 (0.95-0.99) | 1.20 (1.18-1.23) |
| **Pain relief**^a^ |  |  |  |  |
| Epidural anaesthesia | 1.00 | 0.86 (0.84-0.88) | 1.01 (0.99-1.03) | 0.73 (0.71-0.74) |
| **Mode of delivery** |  |  |  |  |
| Elective caesarean^c^ | 1.00 | 1.59 (1.54-1.64) | 1.74 (1.69-1.79) | 1.75 (1.70-1.80) |
| Emergency caesarean^a^ | 1.00 | 1.14 (1.11-1.17) | 1.08 (1.05-1.11) | 0.92 (0.90-0.95) |
| Vacuum^a^ | 1.00 | 1.45 (1.41-1.49) | 1.28 (1.24-1.31) | 1.14 (1.11-1.17) |
| Forceps^a^ | 1.00 | 0.71 (0.68-0.75) | 0.82 (0.79-0.86) | 0.45 (0.43-0.48) |
| Non-operative vaginal^a^ | 1.00 | 0.82 (0.81-0.84) | 0.86 (0.84-0.88) | 1.07 (1.05-1.09) |
| **Select procedures** |  |  |  |  |
| Blood transfusion^c^ | 1.00 | 1.15 (1.06-1.26) | 0.82 (0.75-0.90) | 0.52 (0.47-0.58) |
| Episiotomy^d^ | 1.00 | 0.60 (0.59-0.62) | 0.77 (0.75-0.79) | 0.74 (0.72-0.76) |

^a^ Based on emergency caesarean or vaginal deliveries only (N=286,495)

^b^ Includes medical and surgical methods not elsewhere specified

^c^ Based on all deliveries (N=323,588)

^d^ Based on vaginal deliveries only (N=243,291)
